# Improved Endurance of Ferroelectric Hf_0.5_Zr_0.5_O_2_ Using Laminated-Structure Interlayer

**DOI:** 10.3390/nano13101608

**Published:** 2023-05-11

**Authors:** Meiwen Chen, Shuxian Lv, Boping Wang, Pengfei Jiang, Yuanxiang Chen, Yaxin Ding, Yuan Wang, Yuting Chen, Yan Wang

**Affiliations:** 1 Key Laboratory of Microelectronics Devices and Integrated Technology, Institute of Microelectronics of Chinese Academy of Sciences, Beijing 100029, China; chenmeiwen@ime.ac.cn (M.C.); lvshuxian@ime.ac.cn (S.L.); wangboping@ime.ac.cn (B.W.); jiangpengfei@ime.ac.cn (P.J.); chenyuanxiang@ime.ac.cn (Y.C.); dingyaxin@ime.ac.cn (Y.D.); wangyuan@ime.ac.cn (Y.W.); chenyuting@ime.ac.cn (Y.C.); 2 University of Chinese Academy of Sciences, Beijing 100049, China

**Keywords:** ferroelectrics, ZrO_2_–HfO_2_, interlayer, laminated structure, endurance, reliability, oxygen vacancy

## Abstract

In this article, the endurance characteristic of the TiN/HZO/TiN capacitor was improved by the laminated structure of a ferroelectric Hf_0.5_Zr_0.5_O_2_ thin film. Altering the HZO deposition ratio, the laminated-structure interlayer was formed in the middle of the HZO film. Although small remanent polarization reduction was observed in the capacitor with a laminated structure, the endurance characteristic was improved by two orders of magnitude (from 10^6^ to 10^8^ cycles). Moreover, the leakage current of the TiN/HZO/TiN capacitor with the laminated-structure interlayer was reduced by one order of magnitude. The reliability enhancement was proved by the Time-Dependent Dielectric Breakdown (TDDB) test, and the optimization results were attributed to the migration inhibition and nonuniform distribution of oxygen vacancies. Without additional materials and a complicated process, the laminated-structure method provides a feasible strategy for improving HZO device reliability.

## 1. Introduction

With the emergence of ferroelectricity in doped HfO_2_ films, HfO_2_-based ferroelectric memory has been expected to be a competitive candidate as a next-generation, nonvolatile memory with excellent scalability, low power consumption, fast speed and complementary metal–oxide–semiconductor (CMOS) compatibility [1,2,3,4,5,6,7,8]. The polycrystalline-fluorite-structure HfO_2_ usually behaves as a monoclinic phase at room temperature. With the temperature increasing, there will be transitions among monoclinic, tetragonal and cubic phases [9]. Previous studies revealed that the asymmetric orthorhombic phase with a Pca2_1_ space group is the origin of ferroelectricity [1]. As the thickness decreases through the atomic layer deposition (ALD) process, the tetragonal phases can be more stable at normal temperatures and pressures [10]. For HfO_2_-doped ferroelectric devices, dopants (e.g.: Si, Y, Zr, La, Al, Sr) are used to evoke and stabilize the ferroelectric phase [1,11,12,13,14,15]. Zr dopant is the most commonly used because of the wide doping ratio range and low crystallization temperature which is more compatible with back-end-of-line (BEOL) fabrication [16]. Usually, HZO solid solution films with a HfO_2_ and ZrO_2_ ratio of 1:1 (Hf_0.5_Zr_0.5_O_2_) are chosen to achieve the optimal ferroelectricity [17,18].

However, the endurance characteristic of the HfO_2_-based ferroelectric memory is still an issue to be taken seriously. Because of the destructive reading, the ferroelectric random-access memories are rewritten after each read operation. So, high endurance is necessary for ferroelectric memories. In recent reports, the endurance characteristic of HfO_2_-based ferroelectric devices was lower than that of perovskite-based devices [2,19]. Several methods have been proposed to achieve higher endurance, such as stress control, dopants, temperature regulation and grain boundary interruption. For stress control, the compressive stress using Cu capping applied on HZO thin films was found to achieve high endurance but smaller remanent polarization [20]. Cao et al. found that Ru electrodes can exhibit a lower leakage current and higher breakdown voltage compared with TiN electrodes, which resulted in a higher coercive field [21]. For dopants, Walke et al. demonstrated a high endurance but prolonged wake-up in La and Y doped HZO ferroelectric thin films [22]. For temperature regulation, Choi et al. reported that the different endurance characteristics were observed under different annealing and deposition temperatures [23]. For grain boundary interruption, the most common structure is HZO/Al_2_O_3_/HZO. Xu et al. reported that the grain boundaries penetrating HZO ferroelectric thin films were interrupted by inserting an Al_2_O_3_ layer, and the leakage current was reduced, which could enhance the reliability of HZO devices [24]. However, the optimal grain size is limited by the Al_2_O_3_ interlayer, resulting in small remanent polarization.

Recently, some researchers have focused on the study of thin film deposition methods to adjust the properties of devices. In 2016, Lu et al. reported the induced ferroelectricity of a ZrO_2_/HfO_2_ bilayer, which provided the basis for the emergence of the laminated structure [25]. Subsequently, Weeks et al. reported a laminated structure (1 nm HfO_2_/1 nm ZrO_2_) × 4 which showed the promising ferroelectricity [26]. However, the endurance properties have been less studied in these previous reports. In 2022, Liang et al. studied the performance of a superlattice structure composed of ZrO_2_/HfO_2_ at different annealing temperatures using a ZrO_2_ layer as the starting layer, and reached high endurance performance but low remanent polarization [27]. In 2023, Lehninger et al. studied the performance of HfO_2_/ZrO_2_ superlattices, which showed an obvious wake-up effect, low coercive field, enhanced polarization and high temperature reliability. However, the endurance reached 10^7^ cycles [28]. It is reported that the HfO_2_-starting laminated structure exhibited a higher remanent polarization, and optimal remanent polarization was achieved in a thicker sublayer of about 1 nm [28,29]. The ZrO_2_-starting laminated structure showed a lower remanent polarization, and the remanent polarization decreased with the deposition cycles increasing [30]. However, it also reported that the ZrO_2_ nucleation layer could stabilize the remanent polarization of HZO ferroelectric thin films during field cycling [31]. These previous studies demonstrated the potential of a laminated structure and superlattice structure for improving the ferroelectric and endurance performance.

In this article, we propose a new laminated structure to improve the endurance performance and maintain high remanent polarization. The deposition ratio of the ZrO_2_ and HfO_2_ layers was adjusted with the ZrO_2_ as the starting layer, and the laminated-structure interlayer was formed in the film. The leakage current was reduced by one order of magnitude. The endurance was improved by two orders of magnitude. Furthermore, the breakdown voltage and Time-Dependent Dielectric Breakdown (TDDB) reliability were also enhanced in the TiN/HZO/TiN capacitor with the laminated-structure interlayer. Combined with the migration inhibition and nonuniform distribution of oxygen vacancies, the possible physical mechanisms of laminated structure device performance were analyzed. 

## 2. Materials and Methods

**Figure 1:**
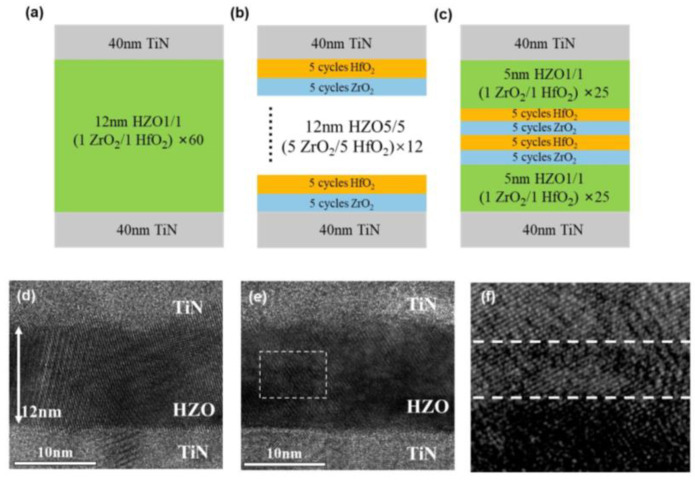
Schematic diagrams of (**a**) the capacitor HZO1/1, (**b**) the capacitor HZO5/5 and (**c**) the capacitor HZO1/5/1. Cross-sectional TEM images of (**d**) the capacitor HZO1/1 and (**e**) the capacitor HZO1/5/1. (**f**): Magnified image extracted from the white box in (**e**).

Figure 1a–c illustrate the structures of three types of TiN/HZO/TiN capacitors. The TiN/HZO/TiN capacitors were fabricated on Si/SiO_2_ substrates. The 40-nm-thick TiN bottom electrodes were deposited by ion beam sputtering. The Hf_0.5_Zr_0.5_O_2_ ferroelectric thin films were deposited by ALD at 280 °C using Hf[N(C_2_H_5_)CH_3_]_4_ (Tetrakis(ethylmethylamido) hafnium, TEMAHf), Zr[N(C_2_H_5_)CH_3_]_4_ (Tetrakis(ethylmethylamido) zirconium, TEMAZr) and H_2_O as an Hf precursor, Zr precursor and oxygen source, respectively. Using ZrO_2_ as the starting layer, the ZrO_2_ and HfO_2_ layers were deposited at the same rate of ~1 Å/cycle. The capacitor HZO1/1 was deposited by alternating 1 cycle of ZrO_2_ and 1 cycle of HfO_2_. Each unit consisted of a ZrO_2_/HfO_2_ bilayer, and the HZO1/1 thin film comprised 60 units with a thickness of 12 nm, as shown in Figure 1a. The capacitor HZO5/5 was deposited by alternating 5 cycles of ZrO_2_ and 5 cycles of HfO_2_, which was composed of 12 units. In addition, the thickness was 12 nm, as shown in Figure 1b. In the case of capacitor HZO1/5/1, first, a 5-nm-thick Hf_0.5_Zr_0.5_O_2_ film was deposited by 1 cycle of ZrO_2_ and 1 cycle of HfO_2_, which was composed of 25 units. Second, the deposition ratio of the ZrO_2_ and HfO_2_ layers was adjusted, and a 2-nm-thick laminated-structure interlayer was deposited. The interlayer was formed by repeating 5 cycles of ZrO_2_ and 5 cycles of HfO_2_ and consisted of 2 units. Third, the first deposition step was repeated for a 5 nm thickness. The total thickness of the HZO1/5/1 capacitor was 12 nm, as shown in Figure 1c. Following the deposition of the HZO thin film, the electrodes were patterned through photolithography on HZO. Subsequently, the 40-nm-thick TiN top electrodes were deposited by sputtering. Finally, all the samples were annealed by rapid thermal annealing (RTA) in a N_2_ atmosphere. The annealing process involved ramping up the temperature at a rate of 8.4 °C/s for 60 s, and the samples were held at a temperature of 500 °C for 30 s.

The electrical characteristics of the capacitors, including the current density–voltage (J–V), dielectric constant–voltage (k–V), polarization–voltage (P–V), and current–time (I–t) curves, were measured using a Keysight B1500 semiconductor parameter analyzer or Radiant Workstation ferroelectric analyzer.

## 3. Results

### 3.1. Basic Electrical Properties

Figure 1d–f show the cross-sectional transmission electron microscopy (TEM) images of capacitors HZO1/1 and HZO1/5/1. The thickness of 12 nm was verified in two capacitors by the TEM images. The interfaces between the TiN electrodes and ferroelectric films were also clearly observed, and the bottom interfaces were clearer due to the sputtering process compared to the top interfaces. In HZO1/1, the lattice arrangement was orderly, and in HZO1/5/1, the laminated-structure interlayer was observed, as seen in Figure 1f,e.

**Figure 2:**
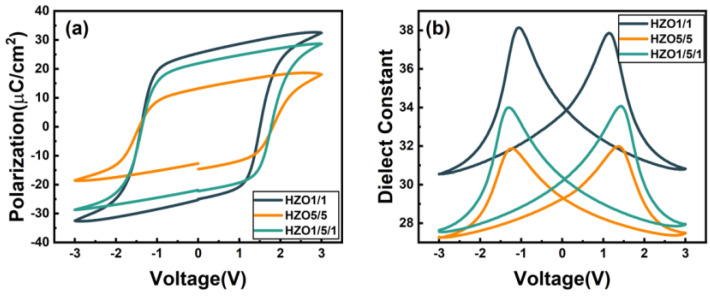
(**a**) P–V loops and (**b**) $\varepsilon_{r}$–V curves of HZO1/1, HZO5/5 and HZO1/5/1 capacitors.

Figure 2a,b show the P–V and $\varepsilon_{r}$–V characteristics of capacitors HZO1/1, HZO5/5 and HZO1/5/1. The P–V loops were obtained after a wake-up cycling of 10^3^ with a pulse voltage of 3 V at 1 kHz. As shown in the P–V loops of Figure 2a, the remanent polarizations (P_r_) of three capacitors were about 25.37 μC/cm^2^, 13.18 μC/cm^2^ and 21.87 μC/cm^2^, respectively. The remanent polarization was slightly reduced in HZO1/5/1 compared with HZO1/1, while the remanent polarization was significantly reduced in HZO5/5. The $\varepsilon_{r}$–V curves of ferroelectric films exhibited typical butterfly-shaped loops at a double sweep voltage from −3 V to 3 V at 100 kHz. The dielectric constants of three capacitors were 33.66, 29.26 and 30.18, respectively. Previous studies have reported the existence of the tetragonal phase and FE orthorhombic phase with a high $\varepsilon_{r}$ value in HfO_2_-based ferroelectric films (T: $\varepsilon_{r}$ = 35–40, O: $\varepsilon_{r}$ = 25–30),and the $\varepsilon_{r}$ value of the monoclinic phase is much lower ($\varepsilon_{r}$ = 15–20) [32]. The lower $\varepsilon_{r}$ value of HZO5/5 and HZO1/5/1 compared to HZO1/1 may reveal a higher M-phase fraction in the HZO5/5 and HZO1/5/1 capacitors.

**Figure 3:**
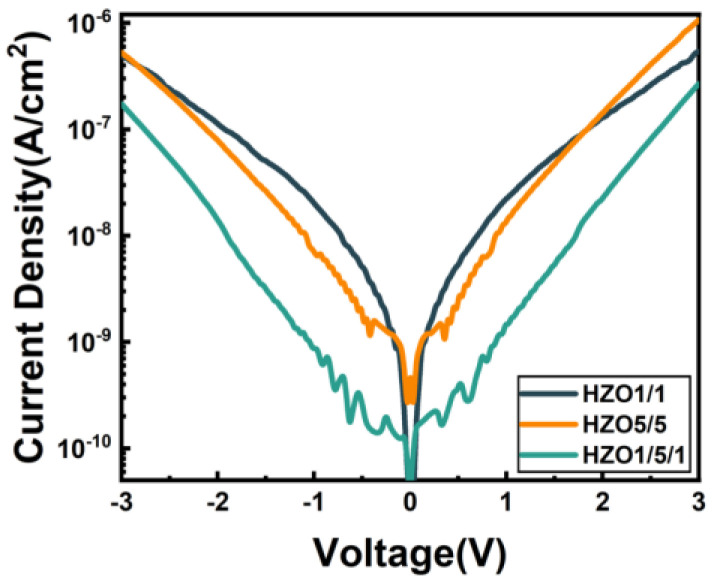
Initial J–V curves of HZO1/1, HZO5/5 and HZO1/5/1 capacitors.

Figure 3 shows the initial J–V curves of three capacitors. The initial leakage current was measured under a sweep voltage from −3 V to 3 V. The initial current densities of HZO1/1 and HZO5/5 were similar. However, compared with HZO1/1, the initial leakage current of the HZO1/5/1 capacitor was reduced by one order of magnitude (from 2.18 × 10^−8^ A/cm^2^ to 1.51 × 10^−9^ A/cm^2^) at a voltage ±1 V. The leakage current was effectively inhibited in HZO1/5/1. The interfaces of the laminated-structure interlayer could prevent the conduction current path [33,34]. 

### 3.2. Endurance and Time-Dependent Dielectric Breakdown Properties

**Figure 4:**
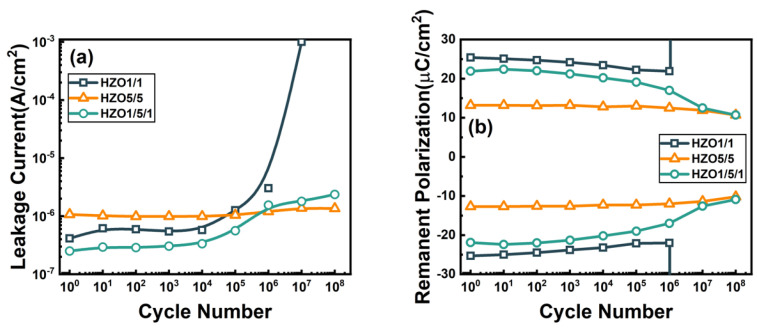
The endurance characteristics of (**a**) leakage currents and (**b**) remanent polarization under cycling pulse with amplitude of ±3 V at 0.1 MHz for HZO1/1, HZO5/5 and HZO1/5/1 capacitors.

Figure 4 presents the endurance performance of three capacitors in terms of the leakage current and the remanent polarization. The cycling test was taken under a triangular pulse with pulse amplitude of ±3 V and pulse frequency of 0.1 MHz. As shown in Figure 4a, the leakage current of HZO5/5 slightly increased from 1.07 × 10^−6^ A/cm^2^ to 1.35 × 10^−6^ A/cm^2^. However, as the pulse cycles increased, the leakage currents obviously increased in the HZO1/1 and HZO1/5/1 capacitors. For HZO1/1, the leakage current sharply increased to 10^−3^ A/cm^2^ after 10^6^ cycles. However, the leakage current increased from 2.51 × 10^−7^ A/cm^2^ to 2.36 × 10^−6^ A/cm^2^ in the HZO1/5/1 after 10^8^ cycles. It can be inferred that the leakage currents during the cycling could be significantly suppressed in the laminated structure, resulting in an improved endurance performance. The remanent polarization of the three capacitors was measured with the cycling test under an electric field of 2.5 MV/cm, as shown in Figure 4b. A hard breakdown was observed in the HZO1/1 when the electric field cycling exceeds ~10^6^ cycles, which is not observed in the HZO5/5 and HZO1/5/1 even up to 10^8^ cycles. The endurance of HZO5/5 and HZO1/5/1 could be improved by two orders of magnitude compared with HZO1/1. The 2P_r_ value of HZO5/5 was slightly reduced from 25.9 μC/cm^2^ to 20.9 μC/cm^2^, and the 2P_r_ value of HZO1/5/1 was reduced from 43.8 μC/cm^2^ to 21.6 μC/cm^2^ up to 10^8^ cycles, but this was still sufficient for memory operation under high cycles. 

**Figure 5:**
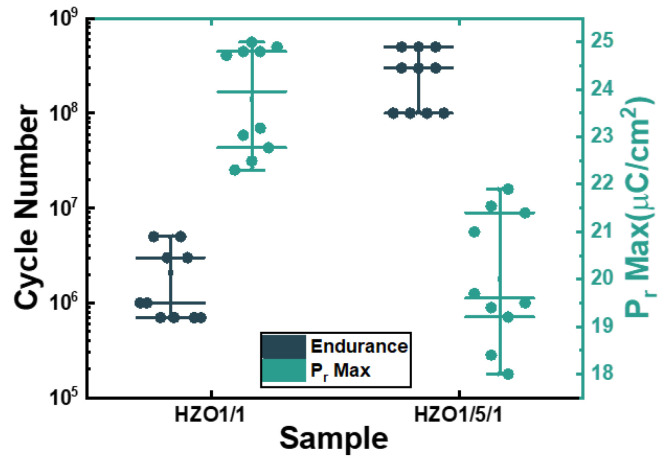
Box chart for endurance and P_r_ max characteristics of HZO1/1 and HZO1/5/1 sample-to-sample variation. Each box plot consists of ten measurements.

Figure 5 shows the statistical chart of the maximum endurance and P_r_ max characteristics of HZO1/1 and HZO1/5/1, where ten measurements were taken for each box to display the sample-to-sample variation. The maximum endurance cycles ranged from 7 × 10^5^ to 5 × 10^6^ for HZO1/1 and 10^8^ to 5 × 10^8^ for HZO1/5/1, indicating that the endurance of HZO1/5/1 was improved by more than two orders of magnitude due to the laminated-structure interlayer. The range of the P_r_ max value was 22.3 μC/cm^2^ to 25 μC/cm^2^ for HZO1/1 and 18 μC/cm^2^ to 21.9 μC/cm^2^ for HZO1/5/1. High remanent polarizations were exhibited in both the HZO1/1 and HZO1/5/1 capacitors.

Figure 4 and Figure 5 demonstrate that the endurance performance of HZO capacitors was effectively improved by the laminated-structure interlayer. The suppression of the leakage current in the HZO1/5/1 was attributed to the prevention of the conduction current path by the interfaces of the laminated-structure interlayer. The high remanent polarization values observed in the improved capacitor HZO1/5/1 suggest its application potential in memory devices.

**Figure 6:**
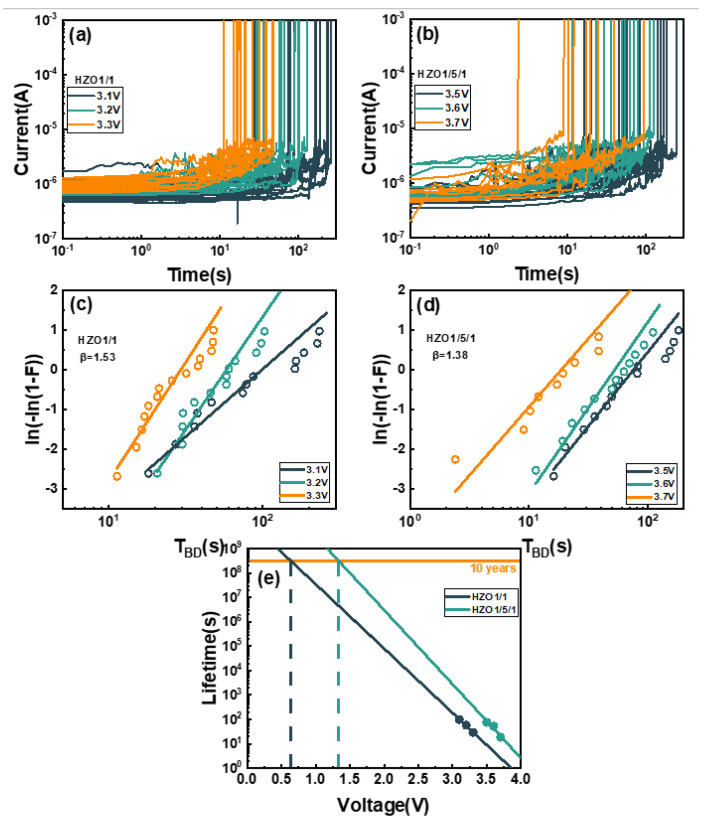
I–t curves of (**a**) the capacitor HZO1/1 and (**b**) the capacitor HZO1/5/1 at three voltages. Weibull distribution of TBD of (**c**) the capacitor HZO1/1 and (**d**) the capacitor HZO1/5/1. (**e**) Ten-year lifetime prediction at 63.2% failure of HZO1/1 and HZO1/5/1 capacitors.

To further investigate the long-term reliability of the HZO1/1 and HZO1/5/1 capacitor, the Time-Dependent Dielectric Breakdown (TDDB) test was utilized with the Constant Voltage Stress (CVS) method. Figure 6a,b show the I–t curves of HZO1/1 and HZO1/5/1 using CVS at three voltages with the area of 1.6 × 10^−5^ cm^2^, and the failure current threshold was set to 1 mA. The hard breakdown could be clearly observed with the top and bottom metal electrodes. Therefore, the breakdown time (T_BD_) was extracted directly. The set voltages of HZO1/1 were 3.1 V, 3.2 V and 3.3 V. For the similar range of the T_BD_ of two capacitors under CVS, the set voltages of HZO1/5/1 were higher by 0.4 V than those of the HZO1/1. As the DC stress voltage increased, the T_BD_ significantly decreasd. This was due to the generation of defects under CVS, which could form conductive paths through the top and bottom electrodes, leading to breakdown [35]. When the leakage current was increased tenfold, the statistics for T_BD_ of the capacitors at three voltages could be established by the Weibull distribution [36]. 

The Weibull plot was applied with cumulative density probability, and the maximum likelihood method was used to fit the data under DC stress, as shown in Figure 6c,d. The cumulative density function in the Weibull plot was given by:

(1) $$W\left(x\right)=Ln(-Ln(1-F\left(x\right)))=\beta Ln\left(x/\alpha \right)$$

where x is the T_BD_, α is the scale-factor, and β is the shape-factor.

The relationship between the operating voltage and lifetime could be obtained by the statistics of the 63.2% failure points in the Weibull distribution, and linearly fitting and extrapolating the DC voltage under the failure point. Figure 6e shows the ten-year lifetime of the two capacitors. For the ten-year lifetime prediction at 63% failure, the operating voltage for HZO1/1 was only 0.64 V, which was much smaller than its coercive voltage. This means that at the operating voltage, the lifetime of HZO1/1 cannot reach ten years. However, the operating voltage for HZO1/5/1 was 1.33 V, which was higher than that for HZO1/1 by 0.6 V. The lifetime of HZO1/5/1 was effectively enhanced compared with that of HZO1/1.

## 4. Discussion

Three capacitors were investigated: ZrO_2_/HfO_2_=1/1 (HZO1/1) was the most common deposition ratio, ZrO_2_/HfO_2_=5/5 (HZO5/5) was the fully laminated structure, and the ZrO_2_/HfO_2_=5/5 laminated structure as an interlayer (HZO1/5/1) was proposed in this work. The electrical test results showed that the remanent polarization was significantly reduced, but the endurance was greatly improved by two orders of magnitude in the fully laminated structure HZO5/5. The advantages of the high endurance of HZO5/5 and high remanent polarization of HZO1/1 were combined in HZO1/5/1. In HZO1/5/1, the endurance was also improved by two orders of magnitude without a significant reduction in the remanent polarization. The internal mechanism of the laminated structure was analyzed in terms of the remanent polarization and endurance performance.

The remanent polarization was an important factor to measure the ferroelectricity of ferroelectric devices. The experimental results showed that the ferroelectric properties of the thin films were affected by changing the ZrO_2_/HfO_2_ deposition ratio. During the crystallization process, the formation of the ferroelectric phase was thought to be the result of inhibiting the tetragonal (T) phase to monoclinic (M) phase transformation, which leads to the formation of the asymmetric orthorhombic (O) phase [1]. Previous studies reported that Hf_0.5_Zr_0.5_O_2_ exhibits optimal ferroelectricity [37]. In the laminated structure, only part of HfO_2_ and ZrO_2_ are completely miscible at the ZrO_2_/HfO_2_ interfaces, and Hf_0.5_Zr_0.5_O_2_ is formed. The incompletely miscible parts of HfO_2_ and ZrO_2_ are shown as less Zr doping in HfO_2_ and more Zr doping in ZrO_2_, respectively. More of the M-phase fraction is shown in less Zr-doped HfO_2_ films, and more of the T-phase fraction is shown in rich Zr-doped HfO_2_ films [17]. The O-phase fraction decreased because of the increase in the T phase or M phase in incompletely miscible parts, which led to the reduction in the remanent polarization of the laminated structure. Therefore, the influence of remanent polarization could be effectively reduced in HZO1/5/1 compared with the fully laminated structure HZO5/5 because the thickness of the laminated structure was small, only 1/6 of the thickness of the whole film.

The leakage current and the breakdown behavior were attributed to the migration and aggregation of defects. Oxygen vacancy is generally regarded as the dominant defect in ferroelectric capacitors. It is reported that the migration barriers from interfaces in HZO and laminated structures are different [38]. The vertical migration of oxygen vacancies can be suppressed in a laminated structure due to the higher energy barriers. For HZO5/5 and HZO1/5/1, since the migration of oxygen vacancies was suppressed in the laminated-structure, the increase in the leakage current was suppressed, resulting in an improved endurance. 

**Figure 7:**
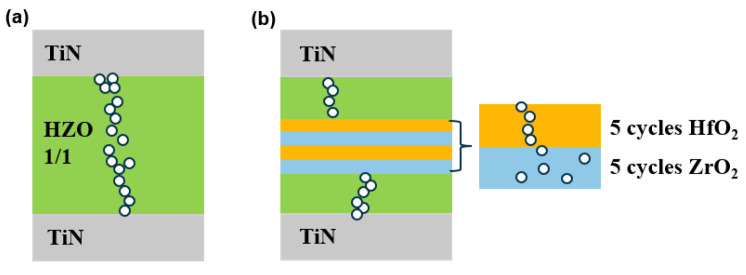
Schematic diagrams of the physical mechanism of the breakdown behavior of (**a**) HZO1/1 and (**b**) HZO1/5/1 devices.

The improved reliability may also be attributed to the nonuniform distribution of defects caused by the laminated-structure interlayer, as shown in Figure 7a,b. The vertical migration and accumulation distribution of the oxygen vacancies promoted the generation and development of conduction in the current path, as shown in Figure 7a. In the HfO_2_ and ZrO_2_ layers, the distributions of the oxygen vacancies were different [39]. The formation of highly conductive filaments in HfO_2_ was accompanied by phase decomposition into hexagonal metal phases, such as Hf and h-Hf_6_O [40]. Compared with ZrO_x_, HfO_x_ had a strong concentration force for oxygen vacancies to produce highly conductive filaments. Under annealing, oxygen vacancies were oriented towards the TiN electrode side, which merged to form conductive filaments where the tip moved towards another TiN electrode side. As shown in Figure 7b, oxygen vacancies tended to accumulate strongly in HfO_2_, but diffusively in ZrO_2_ without obvious accumulation [40]. The non-aggregation of oxygen vacancies in thicker ZrO_2_ layers was difficult for forming a conductive path. Therefore, lower leakage current and higher reliability were achieved in the capacitor with the laminated-structure interlayer.

It is worth noting that the improved endurance of the laminated-structure interlayer was not without negative impacts. The crystallization of the ferroelectric film and the fraction of the phases may have affected the ferroelectric properties of the capacitor. Therefore, it is necessary to carefully balance the improved endurance and the potential negative impact on the ferroelectric properties when using the laminated-structure interlayer. Overall, the analysis of the remanent polarization and endurance provides a deeper understanding of the internal mechanism behind the improved reliability of ferroelectric capacitors with a laminated-structure interlayer.

## 5. Conclusions

In this article, the present study demonstrated the feasibility of using a laminated-structure interlayer as a simple and effective method to improve the endurance of Hf_0.5_Zr_0.5_O_2_ ferroelectric thin films without additional materials and a complicated process. The laminated-structure interlayer was deposited by altering the ZrO_2_–HfO_2_ deposition ratio from 1:1 to 5:5. From the electrical tests and physical characterization, the optimized capacitor exhibited one order of magnitude of reduction in the leakage current and excellent endurance performance improved by two orders of magnitude (from 10^6^ to 10^8^ cycles) compared with HZO1/1. Moreover, the Time-Dependent Dielectric Breakdown (TDDB) reliability was enhanced and the breakdown voltage was increased. These results are ascribed to a different phase fraction, migration inhibition and the nonuniform distribution of oxygen vacancies in the Hf_0.5_Zr_0.5_O_2_ thin film with a laminated-structure interlayer. This work provides a feasible strategy by a deposition ratio adjustment, which contributes to enhancing the reliability of Hf_0.5_Zr_0.5_O_2_ ferroelectric films in nonvolatile memory devices.

## Data Availability

The data presented in this study are available on request from the corresponding author.
